# In patients with mild disability NMOSD: is the alteration in the cortical morphological or functional network topological properties more significant

**DOI:** 10.3389/fimmu.2024.1345843

**Published:** 2024-02-05

**Authors:** Haotian Ma, Yanyan Zhu, Xiao Liang, Lin Wu, Yao Wang, Xiaoxing Li, Long Qian, Gerald L. Cheung, Fuqing Zhou

**Affiliations:** ^1^ Department of Radiology, The First Affiliated Hospital, Jiangxi Medical College, Nanchang University, Nanchang, China; ^2^ Queen Mary College, Nanchang University, Nanchang, China; ^3^ Department of Biomedical Engineering, College of Engineering, Peking University, Beijing, China; ^4^ Spin Imaging Technology Co., Ltd, Nanjing, China; ^5^ Neuroimaging Laboratory, Jiangxi Medical Imaging Research Institute, Nanchang, China

**Keywords:** brain connectomics, neuromyelitis optica spectrum disorder, individual morphological brain network, functional brain networks, topological properties

## Abstract

**Objective:**

To assess the alteration of individual brain morphological and functional network topological properties and their clinical significance in patients with neuromyelitis optica spectrum disorder (NMOSD).

**Materials and methods:**

Eighteen patients with NMOSD and twenty-two healthy controls (HCs) were included. The clinical assessment of NMOSD patients involved evaluations of disability status, cognitive function, and fatigue impact. For each participant, brain images, including high-resolution T1-weighted images for individual morphological brain networks (MBNs) and resting-state functional MR images for functional brain networks (FBNs) were obtained. Topological properties were calculated and compared for both MBNs and FBNs. Then, partial correlation analysis was performed to investigate the relationships between the altered network properties and clinical variables. Finally, the altered network topological properties were used to classify NMOSD patients from HCs and to analyses time- to-progression of the patients.

**Results:**

The average Expanded Disability Status Scale score of NMOSD patients was 1.05 (range from 0 to 2), indicating mild disability. Compared to HCs, NMOSD patients exhibited a higher normalized characteristic path length (λ) in their MBNs (P = 0.0118, FDR corrected) but showed no significant differences in the global properties of FBNs (p: 0.405-0.488). Network-based statistical analysis revealed that MBNs had more significantly altered connections (P< 0.01, NBS corrected) than FBNs. Altered nodal properties of MBNs were correlated with disease duration or fatigue scores (P< 0.05/6 with Bonferroni correction). Using the altered nodal properties of MBNs, the accuracy of classification of NMOSD patients versus HCs was 96.4%, with a sensitivity of 93.3% and a specificity of 100%. This accuracy was better than that achieved using the altered nodal properties of FBNs. Nodal properties of MBN significantly predicted Expanded Disability Status Scale worsening in patients with NMOSD.

**Conclusion:**

The results indicated that patients with mild disability NMOSD exhibited compensatory increases in local network properties to maintain overall stability. Furthermore, the alterations in the morphological network nodal properties of NMOSD patients not only had better relevance for clinical assessments compared with functional network nodal properties, but also exhibited predictive values of EDSS worsening.

## Highlights

- Disrupted global topological properties of mildly disabled NMOSD patients were found only in MBNs.
- In NMOSD patients, disrupted nodal properties were found in both MBNs and FBNs.
- Network-based statistical analysis revealed that MBNs had more significantly altered morphological connections than FBNs.
- Compared to FBNs, MBNs showed better correlations with clinical variables, and better classification efficacy.
- Nodal properties of MBN were predictive of Expanded Disability Status Scale worsening in NMOSD.

## 1 Introduction

Neuromyelitis optica spectrum disorder (NMOSD) is a relatively rare central nervous system autoimmune disease. It is more prevalent in young adults of Asian descent, with a higher incidence among females (1). Clinically, it is characterized by severe optic neuritis (ON) and longitudinally extensive transverse myelitis (LETM). Common lesion sites include the bottom of the fourth ventricle, periependymal gray matter (GM), hypothalamus, walls of the third ventricle, and periventricular regions (1, 2). Patients often experience a prolonged and relapsing course, with the possibility of severe neurological symptoms and even residual neurological impairments (3).

Magnetic resonance imaging (MRI) plays a crucial role in the early identification of NMOSD, guiding aquaporin-4 (AQP4) antibody testing, and aiding in acute-phase treatment decisions. It is also a powerful tool for studying the underlying pathological mechanisms of NMOSD when combined with others novel neuroimaging approaches. For instance, some studies have found that NMOSD patients exhibit increased morphological connectivity in the primary motor modules and motor-sensory related areas of the cerebellum (4), while white matter fiber bundles such as the thalamic radiation, corticospinal tracts, and dorsal and ventral longitudinal fasciculi are damaged (5), leading to decreased local connectivity efficiency (6). However, the alterations in brain structure and function, as well as the mechanisms of plasticity in NMOSD patients, are not fully understood. There are contradictory reports regarding functional connectivity changes, with increased functional connections observed in the default mode network, dorsal attention network, and thalamic network (7), while disease-specific functional connections, such as those in the cerebellar motor modules, show a decrease (4). Additionally, there are conflicting reports of significant functional connectivity increases (8) and decreases in the primary and secondary visual networks (4, 7, 9). Despite local functional connectivity changes, the overall topological properties of the brain tend to remain relatively stable, which is considered a compensatory plasticity mechanism to cope with functional disabilities (10).

The brain is a vast, intricate, and highly efficient operating system composed of billions of neurons. As mentioned earlier, the connectomics, as a tool for studying macroscopic brain white matter structure and functional networks, has been used in NMOSD research (4, 6). However, Pang et al. (11) has demonstrated that the geometry of the brain plays a crucial role in brain function, rather than the connectome eigenmodes. The geometric shape of the brain and its cortical morphological brain networks may provide better insights into the clinical symptoms of NMOSD patients. Indeed, studies on NMOSD patients have found brain cortical atrophy associated with disease progression (12). However, the specific associations between cortical changes and clinical symptoms in NMOSD patients have not been clearly established yet. Individual-based graph cortical morphological brain network analysis is a novel and advanced method that can provide mesoscopic-scale cortical structural information and elucidate its biological significance.

This study hypothesized that cortical geometric changes caused by pathological factors, such as inflammatory lesions, in NMOSD patients can lead to individual cortical morphological brain network (MBN) alterations, which may explain the clinical symptoms of these patients. To explore this hypothesis, this study computed single-subject MBNs based on region similarity methods and applied graph theoretical analysis to investigate the topological properties of constructed networks in NMOSD patients. The resting-state functional brain network (FBN) was also used as a reference for comparison. Correlation analysis was then employed to observe the relationships between network topological properties and clinical variables. Finally, the altered network matrices of topological properties were used to classify NMOSD patients from healthy controls (HCs) and to predictive of disability progression of the patients.

## 2 Materials and methods

### 2.1 Participants

In this study, 21 NMOSD patients and 23 HCs were consecutively recruited at the First Affiliated Hospital of Nanchang University from April 2018 to July 2022. The patients’ inclusion criteria were as follows: (1) met the 2015 international consensus diagnostic criteria of NMOSD; (2) were right-handed and were 18–60 years of age; (3) were AQP4 antibody-positive in serology and/or cerebrospinal fluid status; (4) had not taken drugs such as high-dose steroids for at least 2 weeks before MRI scanning. The exclusion criteria were as follows: (1) a history of other neurological or psychiatric diseases; (2) had contraindications for MRI scans and, (3) image artifacts or incomplete clinical information. In this study, all the NMOSD patients had mild disabilities with EDSS scores of no more than 2 (13, 14).

All of the healthy controls (HCs) were screened using the Clinical Diagnostic Interview Nonpatient Version without significant cognitive disorders, head trauma, or MRI contraindications.

The study was approved by the local human research Ethics Committee and the institutional review board.

### 2.2 Clinical assessment

Each NMOSD patient underwent a comprehensive clinical interview and physical examination, which included the following assessments: (1) the Expanded Disability Status Scale (EDSS) to determine the severity of disability on a scale of 0 to 10. The EDSS has been used to assess the neurological disability of multiple sclerosis, as well as the severity of relapses in patients with NMOSD (14–16), it has validated in NMOSD (16). (2) The Paced Auditory Serial Addition Test (PASAT) to evaluate cognitive function, including auditory information processing speed, flexibility, and calculation ability. (3) The Modified Fatigue Impact Scale (MFIS) to assess the impact of fatigue on daily living.

### 2.3 MRI protocol

The brain images were acquired on a 3.0 Tesla MRI system (Trio, Siemens Healthcare, Erlangen, Germany) with a standard 8‐channel head coil. First, 3-D T1-weighted images were acquired on a magnetization-prepared rapid gradient-echo (MP-RAGE) sequence with the following parameters: repetition time = 1,900 ms, echo time = 2.26 ms, matrix size = 256 × 256, field of view = 240×240 mm, flip angle = 9°, and 176 sagittal slices with thickness = 1.0 mm. Second, resting-state functional MRI (fMRI) were acquired on an echo-planar imaging sequence with the following parameters: repetition time = 2000 ms, echo time = 30 ms, field of view = 200 × 200 mm, flip angle = 90°, 30 axial slices, and interleaved scan with 240 time points. Third, a conventional MRI protocol that included diffusion-weighted imaging (DWI), T2-weighted imaging, and T2-fluid attenuated inversion recovery images was also performed for patient diagnosis and lesion detection. The participants were asked to close their eyes, remain motionless, and avoid systematic thinking and falling asleep during the MRI scan.

### 2.4 Morphological data preprocessing and lesion volume measurement

After manually checking the apparent artifacts, the 3-D T1‐weighted data were preprocessed using the Computational Anatomy Toolbox (CAT12; www.neuro.uni-jena.de/cat/) and run through Statistical Parametric Mapping (SPM12, https://www.fil.ion.ucl.ac.uk/spm/software/spm12/). Each individual T1‐weighted image was segmented into GM, white matter, and cerebrospinal fluid. Brain parenchymal fraction (BPF) was defined as the ratio between the brain parenchymal (GM + white matter) volume and the total volume. Then, GM images were normalized into Montreal Neurological Institute (MNI) 152 space using the Diffeomorphic Anatomical Registration Through Exponential Lie Algebra (DARTEL) modulation approach. Finally, GM images were resliced to a 2 mm isotropic voxel size and spatially smoothed using a Gaussian kernel of 6 mm full-width at half maximum (FWHM).

T2WI lesion volumes were automatically obtained using the Lesion Segmentation Tool (LST) (17) and SPM12 software. Then, the lesion masks in patients were used to fill 3D T1-weighted MR images by the Lesion-Filling Tool (LFT), thereby eliminating the impact of these lesions on brain network construction.

### 2.5 Functional data preprocessing

Resting-state fMRI data were preprocessed using Data Processing & Analysis of Brain Imaging (DPABI, v4.2, http://www.rfmri.org/dpabi) based on SPM12. The standard processing steps (18) included discarding the first 10 time points; slice timing; realignment and correcting (230 remaining time points); coregistering to individual 3-D T1-weighted images; normalization to MNI space; reslicing the functional images (3 × 3 × 3 mm^3^); spatially smoothing with a 6 mm-FWHM Gaussian kernel; detrending and temporal filtering (0.01–0.1 Hz); and nuisance regression (Friston 24-parameter model motion parameters, mean framewise displacement, white matter, cerebrospinal fluid, and global signals).

### 2.6 Network construction

Morphological and functional networks were constructed according to the previous approach (19). First, the nodes of network were defined according to the automated anatomical atlas (AAL) template including 90 cortical and subcortical regions (20).

The edges of network were defined as the interregional similarity based on Kullback–Leibler divergence-based similarity (KLS) measurements for morphological networks (19, 21) and based on interregional linear Pearson correlations for functional networks (22).

### 2.7 Network topological properties analysis

Individual network topological properties were processed using the graph-theoretical network analysis (GRETNA; http://www.nitrc.org/projects/gretna/) toolbox based on 90 × 90 weighted matrices at each sparsity threshold (22). According to previous studies (19, 23), we selected a wide range of sparsity (*S*) thresholds (0.05–0.4) and calculated the area under the curve (AUC) over the *S* thresholds with an interval step of 0.01 for global and nodal topological properties of the MBNs, to avoid potential bias of any arbitrary single threshold selection (21, 24). The global topological properties include the small‐worldness (sigma, *σ*), clustering coefficient (*Cp*) and normalized clustering coefficient (gamma, *γ*), characteristic path length (*Lp*) and normalized characteristic path length (lambda, *λ*), network global efficiency ( $E_{glob}$ ) and local efficiency (*E_loc_*). For global measures, low values of *Lp*, *λ*, and high $E_{glob}$ reflect distributed network integration and the ability for information communication; while high values of Cp , *γ*, and *E_loc_* reflect network segregation, i.e., strong ability of information transfer of interconnected regions. The nodal topological properties included nodal degree, nodal betweenness centrality and nodal efficiency, reflect the topological importance of nodes in the network.

### 2.8 Statistical analysis

The demographic and clinical variables of NMOSD and HC groups were compared using the Statistical Package for the Social Sciences (SPSS) software (version 21.0; IBM Corp., Armonk, New York, USA). Each AUC of the global and nodal topological metrics were compared using nonparametric permutation test (10,000 permutations) for between‐group differences (24, 25), and the Benjamini-Hochberg false discovery rate (FDR q value< 0.05) correction was used for multiple comparisons (24).

Internodal connections with between-group differences in nodal characteristics were compared by a network-based statistical approach (24, 25) (NBS; http://www.nitrc.org/projects/nbs/; corrected at *P*< 0.01).

Partial correlation analysis was performed to examine the relationships between the significantly altered global and/or nodal network properties and clinical variables, controlling for age and sex as confounding variables (Bonferroni correction< 0.05; IBM SPSS Statistics V21.0).

We performed mediation analysis using the SPSS online (https://spssau.com/en/index.html) to further elucidate the relationship among morphological metrics, functional network metrics, and clinical characteristics. In the mediation analysis, total effect of *X* on *Y*(*c*) = indirect effect of *X* on *Y* through *M* (*a*×*b*) + direct effect of *X* on *Y*(*c'*), only variables that showed a significant correlation with others were considered as independent (morphological metrics), dependent (clinical characteristics), or mediating (functional network metrics) variables. The significance analysis was based on 10,000 bootstrap realizations, and age, sex as nuisance variables.

We performed SVM (https://www.csie.ntu.edu.tw/~cjlin/libsvm/) to determine the efficacy of detecting individual NMOSD using the altered network matrices of topological properties (26). 70% of the data were used as the training set to train and the remaining as the test set. The accuracy, sensitivity and specificity of SVM models were calculated. The receiver operating characteristic (ROC) curve and the area under the curve (AUC) were used to evaluate the performance of the models.

Finally, one-way Cox Regression was used for feature screening in order to avoid overfitting. Cox proportional hazards model and Kaplan-Meier survival analysis were used to analyses time-to-progression data.

## 3 Results

### 3.1 Participant demographic and clinical characteristics

Two patients with NMOSD and one HC were excluded due to excessive head motion, and one patient with NMOSD was excluded due to significant data artifacts. Finally, 18 NMOSD patients and 22 healthy participants were included in the comparative study. In the NMOSD patients, the median disease duration was 13 months (2 to 114 months), the median number of relapses was 1 (1 to 4). Four patients had solely spinal lesions, 12 patients had brain lesions (median:1.252 ml; range: 0.016-8.773 ml), including 10 patients with periventricular white matter involvement, 8 patients with centrum semiovale involvement, 6 patients with brain stem or medulla oblongata involvement, 4 patients with area postrema involvement, 2 patients with cerebellum involvement, 1 patient with diencephalon involvement. Seven patients had both spinal cord and brain lesions. The lesion involved the optic nerve alone in 2 patients, and the lesion involved the optic nerve, spinal cord and brain in only 1 patient. There was no significant difference in BPF between patients and controls (*P* = 0.929) (**Table 1**).

**Table 1:** Demographic and clinical characteristics of NMOSD patients and HCs.

Characteristics	NMOSD (n = 18)	HCs (n = 22)	*p*-values
Demographics			
Age at testing (years)	42.110 ± 12.171	44.36±9.052	0.506*
Sex (male/female)	2/16	3/19	0.810#
Handedness (right), %	100	100	1#
Disease-related characteristics			
AQP4-Ab, positive/negative	18/0	n.a.	n.a.
Disease duration (month, median, range)	13 (2-114)	n.a.	n.a.
EDSS scores at baseline	1.056 ± 0.591	0 ± 0	< 0.001
PASAT scores	89.778 ± 14.711	n.a.	n.a.
MFIS scores (median, range)	9 (0-17)	n.a.	n.a.
Mean follow-up time, month (min–max)	39.167 (24-62)	n.a.	n.a.
Follow-up EDSS worsening, n (%)	3 (16.667%)	n.a.	n.a.
MRI-related characteristics			
Lesion volume (ml) (median, range)	1.252 (0.016-8.773)	n.a.	n.a.
Brain parenchymal fraction	0.804 ± 0.024	0.805 ± 0.029	0.929
Framewise displacement (mm)	0.201±0.022	0.188±0.031	0.623*

Data are presented as mean ± standard deviation. # p-value was obtained using the two-tailed chi-squared test; * p-value was obtained by the two-sample two-tailed t-test.

EDSS, Expanded Disability Status Scale; HCs, healthy controls; MFIS, Modified Fatigue Impact Scale; NMOSD, Neuromyelitis optica spectrum disorder; PASAT, Paced Auditory Serial Addition Test.

### 3.2 Global properties of MBNs and FBNs

Based on the predetermined connection density, both the NMOSD patient group and the HC group showed small-world properties in MBNs and FBNs (γ > 1, λ ≈ 1, γ/λ > 1). There were no significant differences in the global properties of FBNs between NMOSD patients and HCs (*P* > 0.05). In contrast, compared with HCs, NMOSD patients showed a significant decrease in *λ* of MBNs (*P* = 0.0118, FDR corrected, effect size: 0.354) but not in other global properties of MBNs (**Table 2**).

**Table 2:** Group comparisons of AUC values of global properties of MBNs and FBNs between NMOSD patients and HCs.

	MBNs			FBNs		
	NMOSD	Control	*p* values	NMOSD	Control	*p* values
*Lp*	0.756 ± 0.024	0.767 ± 0.027	0.1062	0.683 ± 0.028	0.683 ± 0.024	0.488
*Cp*	0.256 ± 0.027	0.257 ± 0.003	0.3737	0.251 ± 0.014	0.252 ± 0.008	0.446
*γ*	0.502 ± 0.003	0.494 ± 0.005	0.1824	0.635 ± 0.084	0.631 ± 0.066	0.418
*λ*	0.417 ± 0.005	0.419 ± 0.026	0.0118*	0.423 ± 0.012	0.423 ± 0.008	0.487
*σ*	0.481 ± 0.025	0.470 ± 0.026	0.1014	0.596 ± 0.081	0.590 ± 0.067	0.406
*E_glob_*	0.224 ± 0.026	0.222 ± 0.003	0.1802	0.243 ± 0.006	0.242 ± 0.006	0.405
*E_loc_*	0.301 ± 0.003	0.301 ± 0.004	0.4523	0.316 ± 0.008	0.316 ± 0.005	0.408

Permutation tests were used to determine the differences in the global network properties between groups (see Materials and Methods). Values were the fitted AUC values (mean ± SD) of global network properties in each group. *p< 0.05.

Cp = clustering coefficient; Lp = characteristic path length; Gamma = normalized clustering coefficient; Lambda = normalized characteristic path length; Sigma = small-worldness; Eglob = network global efficiency; Eloc = local efficiency; FBNs, functional brain networks; HCs, healthy controls; MBNs, morphological brain networks. The same abbreviations are used in the other figures and tables; therefore, this note is not repeated.

### 3.3 Nodal properties of MBNs and FBNs

**Figure 1:**
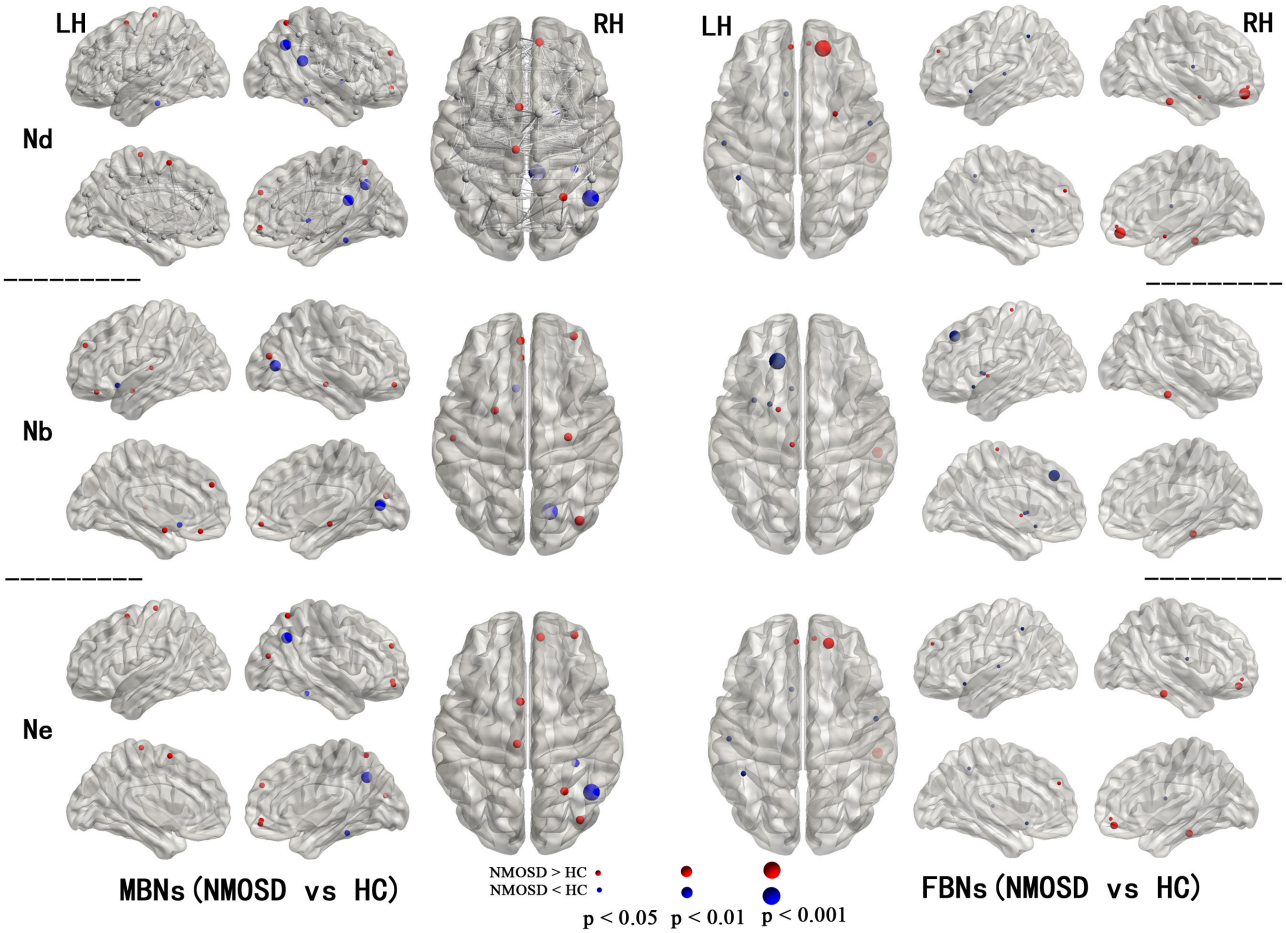
Regions with significant alterations in the nodal properties of MBNs and FBNs between NMOSD patients and healthy subjects (*P*< 0.05, FDR corrected). All the brain regions were defined by AAL-90; see **Supplementary Tables S1**, **S2** for the full names of the nodes in the figure.

For node centrality measures of MBNs and FBNs, significant differences are shown in **Figure 1** (**Supplementary Tables S1**, **S2**) between patients with NMOSD and HCs (*P*< 0.05, FDR corrected). In NMOSD patients, except for the left olfactory cortex, both MBN and FBN showed a decrease in nodal betweenness (Nb), but there was no overlap in other nodal properties that decreased in brain regions. Among the brain regions where nodal properties were increased in NMOSD patients, the MBN exhibited more compensatory increases in brain areas associated with motor, visual, and emotional functions, such as the motor network (MON), visual I network (VisI), frontoparietal network (FPN), and medial frontal network (MFN), than the FBN.

### 3.4 Alterations in the connection of MBNs and FBNs

**Figure 2:**
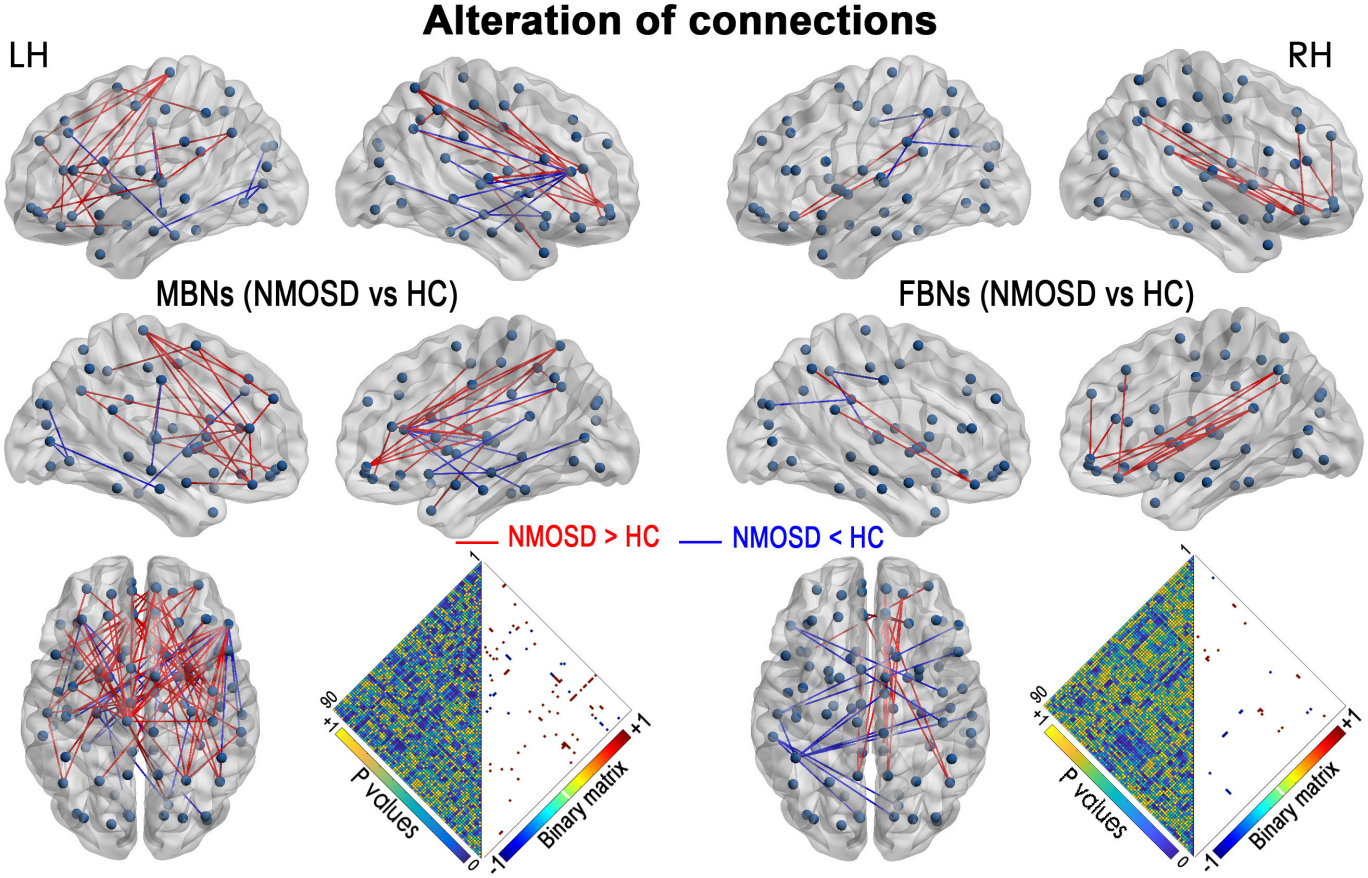
NMOSD-related alterations in the network connections in MBNs and FBNs (P< 0.01, NBS corrected). Each node denotes a brain region of AAL-90. Significantly decreased connections in patients with NMOSD compared with HCs are presented in blue, and increased connections are presented in red. Heatmaps show the P values between the nodes and the edges with significant alterations (binary matrix). LH, left hemisphere; RH, right hemisphere.

Compared with HCs, both MBNs and FBNs in NMOSD patients had increased and decreased network connectivity (P< 0.01, NBS corrected) (**Figure 2**). For MBNs, significantly increased connections involving 54 nodes and 74 edges, as well as decreased connections involving 23 nodes and 23 edges, were observed in patients compared with HCs. For FBNs, there were fewer significant alterations in the connections in patients compared to MBNs, with significantly increased connections involving 16 nodes and 18 edges and decreased connections involving 16 nodes and 13 edges, respectively, compared with HCs.

### 3.5 Relationships between global network properties and clinical variables

**Figure 3:**
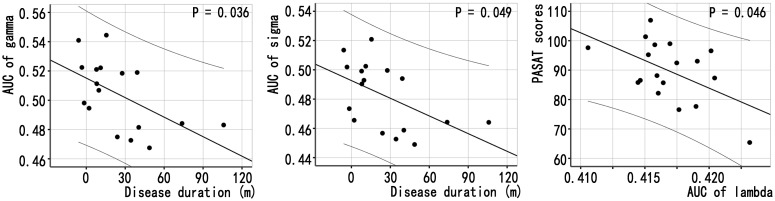
Associations between global properties and clinical variables in patients with NMOSD. Scatter plots of global properties of MBNs and clinical variables.

The relationships between the global network properties of patients with NMOSD and clinical variables were shown in **Figure 3** and **Supplementary Tables S3**, **S4**: (1) disease duration was correlated with the gamma (γ) AUC (*P* = 0.036, without correction), the sigma (σ) AUC (*P* = 0.049, without correction) of MBNs; and (2) the lambda (*λ*) AUC of MBNs was correlated with PASAT scores (*P* = 0.049, without correction). There was no significant correlation between global properties of functional networks and clinical variables.

### 3.6 Significant relations between nodal properties and clinical variables

**Figure 4:**
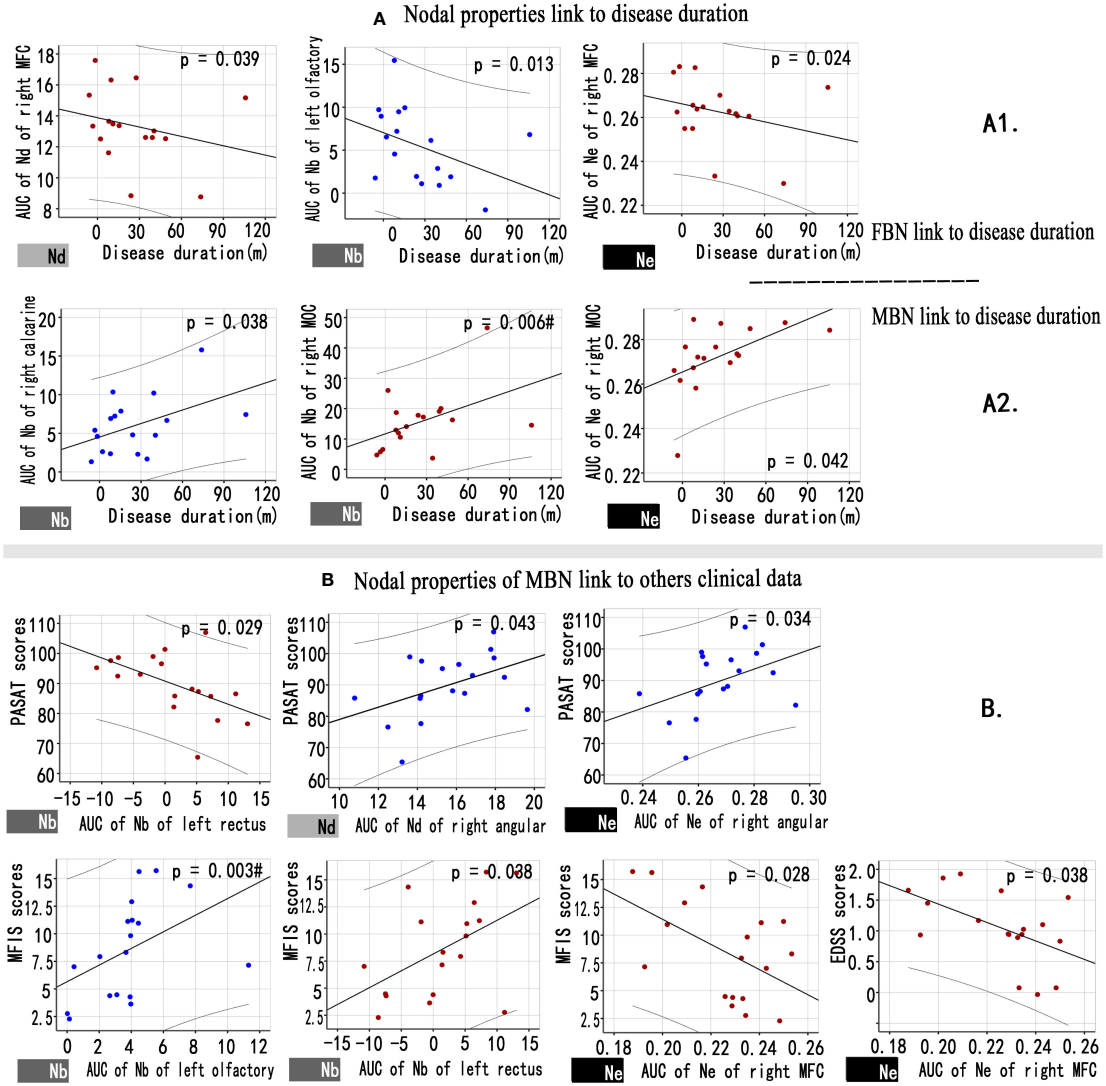
Associations between nodal properties and clinical variables in patients with NMOSD. **(A)** Scatter plots of disease duration and the altered FBN (A1), MBN (A2) nodal properties. **(B)** Scatter plots of the altered MBN nodal properties and others clinical variables. Blue points represent attributes that decreased nodal properties in the patient group compared to the control group, while red points represent attributes that increased nodal properties in NMOSD patients. # Bonferroni correction P< 0.05/6. FBN, functional brain network; MBN, morphological brain network; Nd, nodal degree; Nb, nodal betweenness; Ne, nodal efficiency.

**Figure 4** showed a correlation between the nodal properties of MBNs or FBNs and clinical variables in patients with NMOSD (*P*< 0.05; see **Supplementary Tables S5**, **S6** for details). After Bonferroni correction, the disease duration was significantly correlated with the Nb AUC of right middle occipital gyrus (MOG) of MBNs (*P* = 0.006< 0.05/5); and the Nb AUC of left olfactory cortex of MBNs was significantly correlated with the MFIS scores (*P* = 0.003< 0.05/5).

### 3.7 Mediation analysis and single‐subject classification

In NMOSD patients, mediation analysis did not find any mediating effect between morphological network topological properties, functional network topological properties, and clinical parameters.

**Figure 5:**
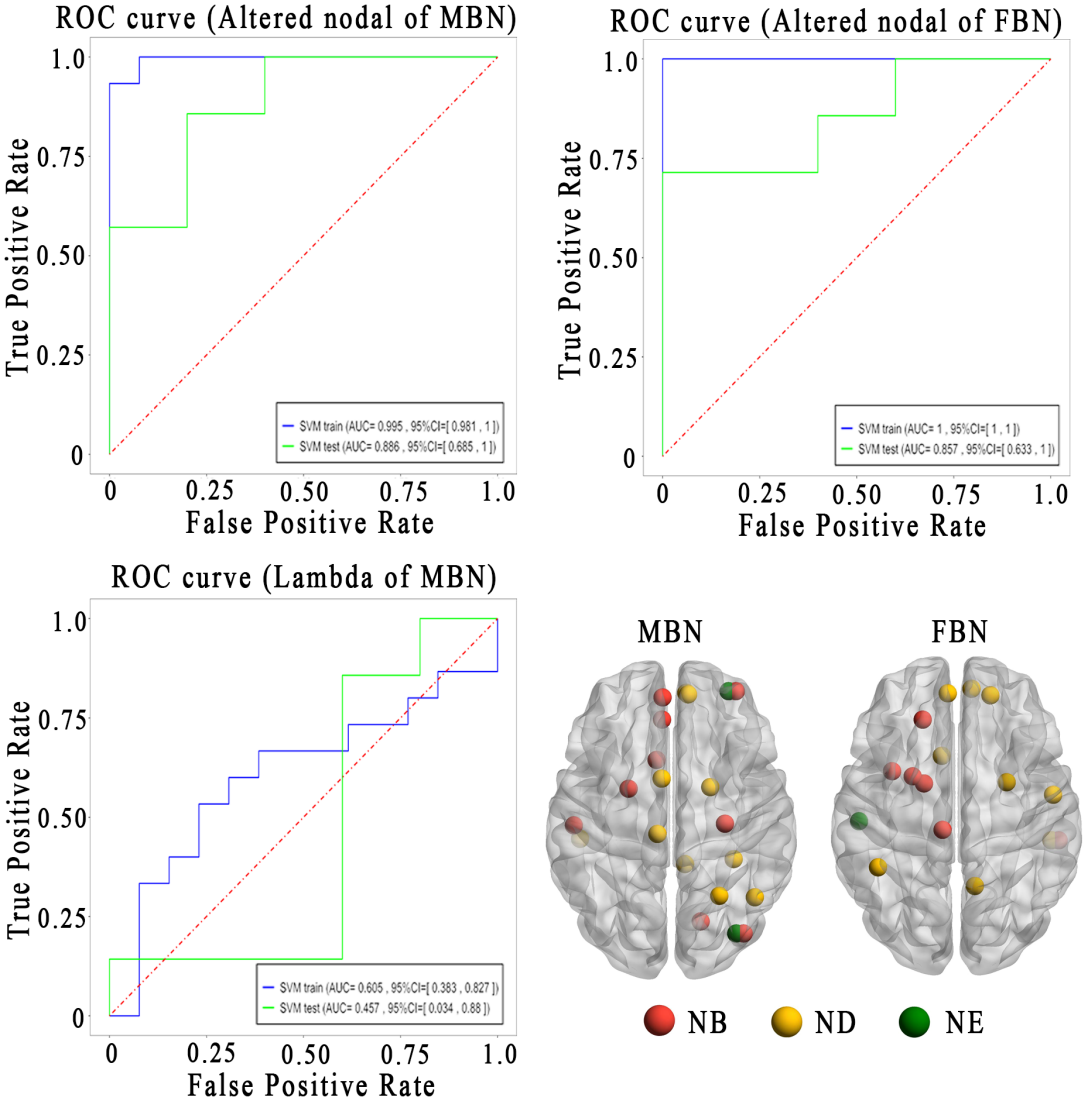
Sensitivity and specificity of the altered brain network topological properties in differentiating the patients with NMOSD from HCs. Lower left is the altered brain regions for the classification analysis. AUC, area under the curve; FBN, functional brain network; MBN, morphological brain network; ROC, receiver operating characteristic curves; Nb, nodal betweenness; Nd, nodal degree; Ne, nodal efficiency.

Using the altered nodal properties of MBNs, the accuracy of classifying NMOSD patients versus HCs was 96.4%, with a sensitivity of 93.3% and a specificity of 100%. Using FBNs, the accuracy was 85.7%, with a sensitivity of 73.3% and specificity 100% (**Figure 5**; **Supplementary Table S7**). The relevant nodal properties contributing to the SVM classification are shown in **Supplementary Table S7**. However, when using the altered global properties of MBNs (*λ*), the discrimination of NMOSD patients from HCs at the single-subject level was insufficient. The accuracy was only 64.3%, with a sensitivity of 53.3% and a specificity of 76.9%.

**Figure 6:**
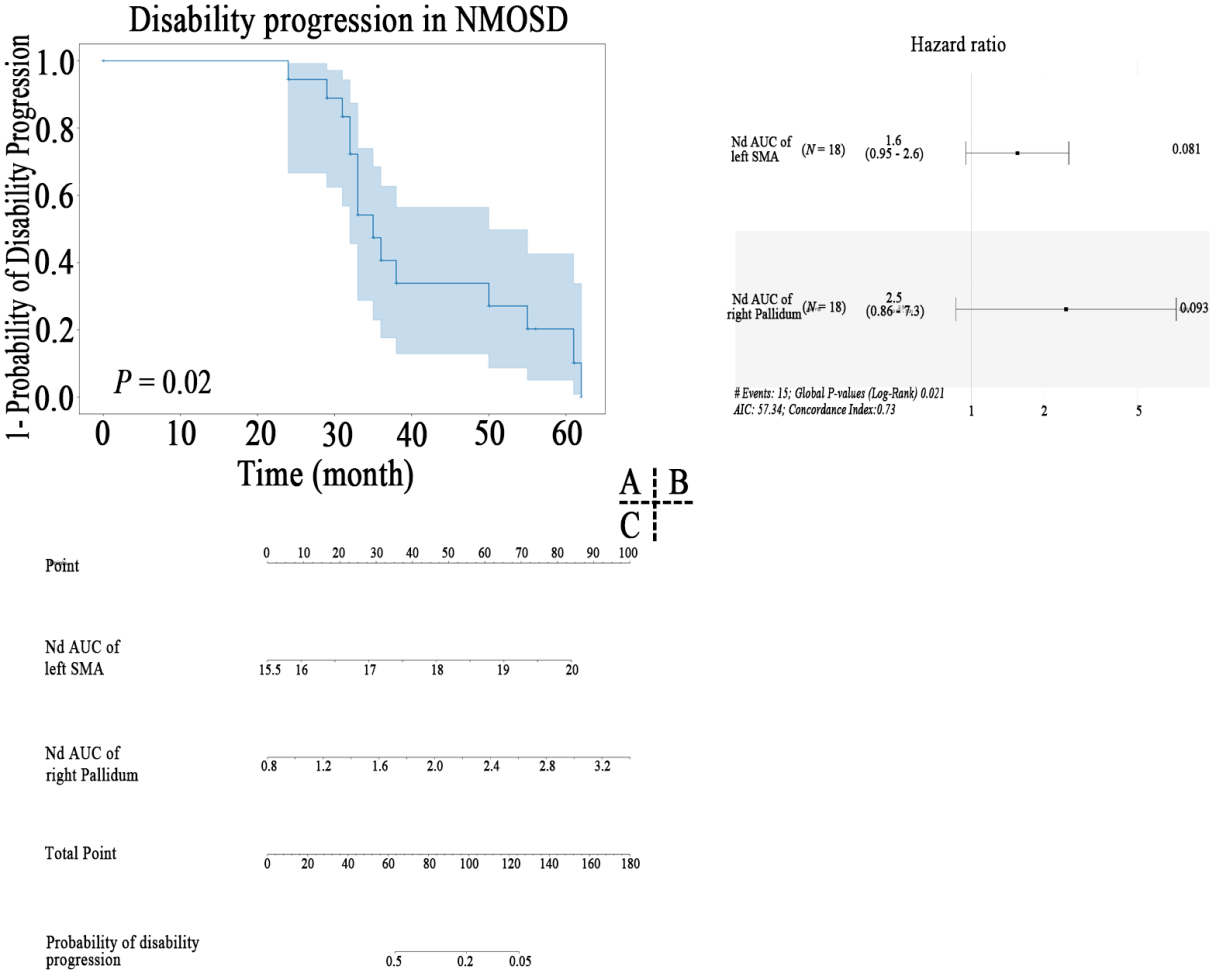
Kaplan–Meier survival curve of nodal properties of MBN predicting disability progression in patients with NMOSD **(A)**, the results of the multivariate Cox regression **(B)**, and nomogram **(C)** which were constructed by the significant features.

Kaplan-Meier survival analysis indicated that nodal properties of MBN were predictive of progression in NMOSD patients (**Figure 6A**). After feature screening by univariate Cox Regression, there were two significant features remaining among the 28 features (**Figure 6B**), namely Nd AUC of left SMA (*P* = 0.022) and Nd AUC of right pallidum (*P* = 0.020). In a univariable model, Nd AUC of left SMA of MBN (HR=1.56 (95% CI 0.947 to 2.56) p=0.081) and Nd AUC of right pallidum (HR=2.50 (95% CI 0.859 to 7.26) p=0.093) were trend associated with EDSS worsening in patients with NMOSD. Cox proportional hazards model to evaluate their prognostic values. Based on the model, a nomogram was established to predict the time-to-progression (**Figure 6C**).

## 4 Discussion

In this study, we first investigated the alterations in MBNs in NMOSD patients and their correlations with clinical variables. (1) The MBNs showed a decrease in lambda that was associated with PASAT scores, indicating a reduced overall efficiency of interregional information integration in NMOSD patients with mild disability. However, the FBNs did not reveal such changes. (2) Compensatory increases in nodal properties, including visual-related networks, MONs, and medial frontal networks (MFNs), contributed to maintaining the stability of global properties in the MBNs of NMOSD patients. (3) Nodal properties of the VisI and the visual association network (VA) were correlated with disease duration, while nodal properties of MFN, FPN, and DMN were correlated with clinical symptom assessment. (4) Nodal properties of MBN were predictive of EDSS worsening in patients with NMOSD, suggesting its additional clinical value as a non-invasive biomarker for mild disability patients with NMOSD. This study provides a new perspective that the properties of local nodes, such as the visual network of MBNs, rather than FBNs, may play a key role in patients with NMOSD.

### 4.1 Altered global properties of brain networks in NMOSD patients

We found that NMOSD patients exhibited a decrease in the global efficiency of interregional information integration in MBNs, which was significantly correlated with PASAT scores. However, such alterations were not observed in FBNs. NMOSD patients often experience fatigue, poor sleep quality, and cognitive impairments characterized by decreased processing speed, executive function, and memory, with processing speed decline being particularly prominent (27). Studies on brain white matter structural networks in NMOSD patients have also shown disruptions in white matter fiber connections, which are associated with declines in cognitive functions related to attention, working memory, processing speed, and visuospatial processing (28). The characteristic path length reflects the global feature of a network, and a smaller value indicates faster information transfer within the network. In our study, we observed a mild decrease in lambda in NMOSD patients, which was negatively correlated with the decline in PASAT scores, suggesting that compensatory mechanisms in certain NMOSD patients may help slow the decline in processing speed. This finding may be related to the fact that NMOSD patients in our study had mild disability and mild cognitive impairment.

### 4.2 Altered nodal properties of brain networks in NMOSD patients

First, nodal property decreases are straightforward, as they are related to inflammatory white matter lesions (5, 29) and network disconnection (28, 30), resulting in corresponding clinical functional impairments (31). The NMOSD patients in our study experienced fatigue, mild disability, and mild cognitive impairment, which are common clinical symptoms of NMOSD (1, 27, 32). However, the global network properties of patients remained relatively stable, which is related to the observed local increases. In our study, the areas with increased nodal properties were mainly found in the following regions: (1) the MFN. This network is highly implicated in the modulation of internal emotional stimuli and automatic emotional responses. Nodes right medial superior frontal cortex (AAL24) and left rectus gyrus (AAL27) of MFN were correlated with cognitive (PASAT) and fatigue (MFIS) assessments. With the exacerbation of symptoms, NMOSD patients showed increased degree and betweenness centrality, indicating the presence of pseudoadaptive compensation. Similar findings have also been reported in studies of multiple sclerosis (33); (2) the MON and VA. These regions are related to motor and visual functions. Node right middle occipital gyrus (AAL52) of the VA and disease duration were correlated, and in combination with the correlation between decreased nodal properties (VisI) and disease duration, it indicates that compensatory mechanisms for visual processing in response to the functional decline in the visual I region of NMOSD patients gradually increase. Whether this compensation can effectively improve the clinical symptoms of patients requires further research in the future; (3) the default mode network (DMN). DMN does not directly affect the MON or directly correlate with fatigue but is related to motor preparation. The dynamic interaction between the default network and MON may affect motor task execution abilities (34). Highly active nodes of the default network consume excessive energy, leading to a state of “idleness” fatigue. Similar phenomena have also been found in studies of multiple sclerosis, where a highly active DMN was correlated with fatigue (35). In our study, the efficiency of DMN nodes increased and was negatively correlated with disability level (EDSS scores) and fatigue (MFIS scores), indicating that the default network nodes (AAL26) of NMOSD indirectly contributed to the aforementioned functional changes; and (4) the frontoparietal network (FPN). FPN rapidly accomplishes new tasks through flexible interactions with other control and processing networks. This may be the reason no correlation was found between these brain areas and clinical data in this study. Last, compensatory mechanisms are not uncommon in NMOSD. For example, a study on cerebellar connectivity in NMOSD patients found that increased connectivity in the primary motor module of the cerebellum can reflect specific cortical injury and serve as compensation for patients’ motor and sensory functions (4).

In the SVM analysis, we achieved a higher (96.4%) classification accuracy using altered nodal properties of MBNs, compared to the classification accuracy (85.7%) of using FBNs, showing that altered nodal properties of MBN were more sensitive in identifying NMOSD from HCs. Our findings support the emerging view that cortical morphological networks, which are crucial in brain function (36),are powerful tools for examining the structural reorganization due to inflammatory and demyelinating damage (33). The morphological network biomarkers have the potential to improve the diagnosis of neurological diseases. Moreover, the altered nodal properties of MBN were directly related to clinical variables, and this relationship was not mediated by functional nodal properties. This underscores the greater sensitivity and clinical significance of topological properties of MBNs compared to functional parameters in NMOSD. These findings bolster the concept that nodal properties of morphological network might serve as a neuroimaging biomarker to assist the clinical diagnosis of mildly disabled NMOSD in the future (12, 33, 36, 37).

Furthermore, in the MBN, increased Nd of left SMA and decreased Nd of right pallidum was predictive of EDSS worsening in NMOSD, which is in line with the idea that brain connectional morphological feature is a composite marker of ageing and a disease-related brain (38). The increased node degree of left SMA side predicts the EDSS worsening, further indicating the presence of pseudoadaptive compensation of supplementary motor area, which is consistent with the hypothesis of the increase of node attributes discussed above. In a recent study of deep learning-derived brain age gap base on morphological MRI, the authors reported brain age gap (5.4 (95% CI 4.3 to 6.5) years) significantly predicted EDSS worsening in patients with NMOSD in the 6 tertiary neurological centers of China cohort (39). Future studies are required to determine the possible causative EDSS worsening factors in individual-scale parameters.

### 4.3 Limitations

This study had several limitations. First, there was no distinction between patients in the acute phase and the remission phase, and different disease stages or phenotypes may have an impact on brain network topological properties. However, all NMOSD patients in this study had mild disability and mild cognitive impairment. This may explain why the global network properties of the patients remained relatively normal. In future research, it will be important to consider the influence of different disease stages or phenotypes on brain network topological properties. Second, different segmentation templates or network nodes may affect the calculation and comparison of patients’ network topological properties. Previous studies have found high consistency between the Harvard-Oxford atlas templates and AAL templates (40). This study also compared different segmentation templates and node correlation methods, and similar results were obtained. Moreover, previous methodological studies have demonstrated their good reproducibility (21, 41).

## 5 Conclusions

This study suggested that NMOSD patients in a mildly disabled state exhibited compensatory increases in local topological properties to maintain the overall stability of the brain network. Compared to functional networks, the nodal properties of MBNs not only revealed more alterations in NMOSD patients but also showed better correlations with clinical assessments and predicted EDSS worsening.

## Data availability statement

The raw data supporting the conclusions of this article will be made available by the authors, without undue reservation.

## Ethics statement

The studies involving humans were approved by Medical Ethics Committee of the First Affiliated Hospital of Nanchang University. The studies were conducted in accordance with the local legislation and institutional requirements. The participants provided their written informed consent to participate in this study.

## Author contributions

HM: Writing – original draft, Investigation, Formal analysis, Funding acquisition, Visualization. YZ: Writing – review & editing, Data curation, Formal analysis, Visualization. XL: Data curation, Formal analysis, Writing – review & editing. LW: Data curation, Formal analysis, Writing – review & editing. YW: Data curation, Writing – review & editing. XXL: Data curation, Writing – review & editing. LQ: Data curation, Writing – review & editing. GC: Data curation, Writing – review & editing. FZ: Resources, Software, Supervision, Validation, Visualization, Conceptualization, Data curation, Funding acquisition, Writing – original draft.
