# Supplementary material for: In patients with mild disability NMOSD: is the alteration in the cortical morphological or functional network topological properties more significant
Source: Front Immunol. 2024 Feb 5;15:1345843. doi: 10.3389/fimmu.2024.1345843 (PMC10875087; doi:10.3389/fimmu.2024.1345843)
Supplement: Supplementary file 1 [file DataSheet_1.docx]

**SUPPLEMENTARY MATERIALS**

**Table S1.** Comparison of nodal properties of MBNs between NMOSD patients and HCs.

| Brain regions | BA | P values | | | | Yale Networks | AAL |  |
| --- | --- | --- | --- | --- | --- | --- | --- | --- |
|  |  | Nodal degree | Nodal betweenness | | Nodal efficiency |  |  |  |
| NMOSD > HC | | | | | | | | |
| Frontal_Mid_Orb_R | 10 | 0.102 | 0.028* | 0.048* | | Frontoparietal network (FPN) | 10 | |
| Supp_Motor_Area_L | 6 | 0.039* | 0.165 | 0.018* | | Medial frontal network (MFN) | 19 | |
| Frontal_Sup_Medial_L | 10 | 0.511 | 0.038* | 0.405 | | Medial frontal network (MFN) | 23 | |
| Frontal_Sup_Medial_R | 10 | 0.042* | 0.086 | 0.022* | | Medial frontal network (MFN) | 24 | |
| Frontal_Med_Orb_R | 10 | 0.032* | 0.132 | 0.010* | | Default mode network (DMN) | 26 | |
| Rectus_L | 11 | 0.462 | 0.015* | 0.381 | | Medial frontal network (MFN) | 27 | |
| Hippocampus_R | 54 | 0.466 | 0.049* | 0.326 | | Basal Ganglia | 38 | |
| Amygdala_L | 53 | 0.438 | 0.036* | 0.351 | | Motor network (MON) | 41 | |
| Occipital_Mid_R | 19 | 0.078 | 0.018* | 0.034* | | Visual association network (VA) | 52 | |
| Parietal_Sup_R | 7 | 0.043* | 0.155 | 0.036* | | Visual association network (VA) | 60 | |
| Paracentral_Lobule_L | 4 | 0.037* | 0.143 | 0.029* | | Motor network (MON) | 69 | |
| Temporal_Sup_L | 22 | 0.080 | 0.033* | 0.072 | | Motor network (MON) | 81 | |
| NMOSD < HC | | | | | | | | |
| Olfactory_L | 25 | 0.123 | 0.039* | | 0.215 | Basal Ganglia | 21 |  |
| Cingulum_Post_R | 23 | 0.003** | 0.090 | | 0.112 | Default mode network (DMN) | 36 |  |
| Calcarine_R | 17 | 0.059 | 0.005** | | 0.054 | Visual I network (VisI) | 44 |  |
| Fusiform_R | 37 | 0.029* | 0.102 | | 0.029* | Visual Association | 56 |  |
| Angular_R | 39 | 0.003** | 0.274 | | 0.008** | Frontoparietal network (FPN) | 66 |  |
| Pallidum_R | 51 | 0.024* | 0.316 | | 0.303 | Basal Ganglia | 76 |  |
| Temporal_Inf_L | 20 | 0.049* | 0.309 | | 0.085 | Frontoparietal network (FPN) | 89 |  |

Note:  Benjamini‐Hochberg false discovery rate corrected. All nodes have at least one type of significant nodal property change. * P < 0.05, **P < 0.01, ***P < 0.001. All the brain regions were defined by AAL atlas. Yale Networks: Networks defined on the Shen 268 atlas.

Abbreviations: AAL, Automated anatomical atlas; BA, Brodman areas.

**Table S2**. Comparison of nodal properties of FBNs between NMOSD patients and HCs.

| Brain regions | BA | P values | | | | Yale Networks | | AAL |  |
| --- | --- | --- | --- | --- | --- | --- | --- | --- | --- |
|  |  | Nodal degree | Nodal betweenness | Nodal efficiency | |  |  |  | |
| NMOSD > HC | | | | | | | | |  |
| Frontal_Sup_Orb_R | 11 | 0.0007*** | 0.053 | 0.001** | Frontoparietal network (FPN) | | 6 | | |
| Frontal_Sup_Medial_L | 10 | 0.020* | 0.207 | 0.029* | Medial frontal network (MFN) | | 23 | | |
| Frontal_Med_Orb_R | 10 | 0.031* | 0.372 | 0.038* | Default mode network (DMN) | | 26 | | |
| Amygdala_R | 53 | 0.021* | 0.417 | 0.071 | Motor network (MON) | | 42 | | |
| Paracentral_Lobule_L | 4 | 0.143 | 0.030* | 0.171 | Motor network (MON) | | 69 | | |
| Pallidum_L | 51 | 0.181 | 0.046* | 0.202 | Basal Ganglia | | 75 | | |
| Temporal_Inf_R | 20 | 0.007** | 0.008** | 0.007** | Frontoparietal network (FPN) | | 90 | | |
| NMOSD < HC | | | | | | | | |  |
| Frontal_Sup_L | 6 | 0.321 | 0.0003*** | 0.253 | | Medial frontal network (MFN) | | 3 | |
| Rolandic_Oper_R | 6 | 0.036* | 0.220 | 0.035* | | Motor network (MON) | | 18 | |
| Olfactory_L | 25 | 0.015* | 0.045* | 0.011* | | Basal Ganglia | | 21 | |
| Insula_L | 13 | 0.077 | 0.023* | 0.070 | | Limbic system | | 29 | |
| Parietal_Inf_L | 40 | 0.036* | 0.426 | 0.021* | | Frontoparietal network (FPN) | | 61 | |
| Putamen_L | 49 | 0.192 | 0.036* | 0.291 | | Basal Ganglia | | 73 | |
| Temporal_Sup_L | 22 | 0.042* | 0.114 | 0.039* | | Motor network (MON) | | 81 | |

Note: Benjamini‐Hochberg false discovery rate corrected. All nodes have at least one type of significant nodal property change. * P < 0.05, **P < 0.01, ***P < 0.001. All the brain regions were defined by AAL atlas. Yale Networks: Networks defined on the Shen 268 atlas.

Abbreviations: AAL, Automated anatomical atlas; BA, Brodman areas.

**Table S3.** Correlation analysis between global properties of MBNs of NMOSD patients and clinical assessments (with age and gender as covariates).

|  | | DD | BPF | EDSS | PASAT | MFIS | MMSE |
| --- | --- | --- | --- | --- | --- | --- | --- |
| AUC of $Lp$ | Correlation | -0.006 | -0.360 | 0.455 | -0.225 | 0.366 | -0.129 |
|  | P values | 0.983 | 0.171 | 0.102 | 0.440 | 0.198 | 0.646 |
| AUC of $Cp$ | Correlation | -0.125 | 0.318 | -0.220 | 0.222 | -0.246 | 0.165 |
|  | P values | 0.671 | 0.229 | 0.449 | 0.445 | 0.396 | 0.557 |
| AUC of $\gamma$ | Correlation | -0.562 | 0.394 | 0.343 | 0.076 | -0.192 | 0.335 |
|  | P values | 0.036* | 0.131 | 0.229 | 0.798 | 0.510 | 0.223 |
| AUC of $\lambda$ | Correlation | -0.123 | 0.223 | 0.445 | -0.541 | 0.495 | 0.006 |
|  | P values | 0.676 | 0.406 | 0.111 | 0.046* | 0.072 | 0.982 |
| AUC of $\sigma$ | Correlation | -0.534 | 0.362 | 0.273 | 0.16 | -0.263 | 0.335 |
|  | P values | 0.049* | 0.168 | 0.346 | 0.584 | 0.364 | 0.223 |
| AUC of $E_{glob}$ | Correlation | -0.025 | 0.405 | -0.397 | 0.054 | -0.160 | 0.097 |
|  | P values | 0.932 | 0.120 | 0.159 | 0.856 | 0.585 | 0.730 |
| AUC of $E_{loc}$ | Correlation | -0.195 | 0.395 | -0.090 | 0.311 | -0.293 | 0.297 |
|  | P values | 0.504 | 0.130 | 0.759 | 0.279 | 0.309 | 0.282 |

Note: * p<0.05 ** p<0.01.

Abbreviations: BPF, Brain parenchymal fraction; DD, Disease duration; EDSS, Expanded Disability Status Scale; PASAT, Paced Auditory Serial Addition Test; MFIS, Modified Fatigue Impact Scale; MMSE, Mini-Mental Status Exam.

**Table S4.** Correlation analysis between global properties of FBNs of NMOSD patients and clinical assessments (with age and gender as covariates).

|  | | DD | BPF | EDSS | PASAT | MFIS | MMSE |
| --- | --- | --- | --- | --- | --- | --- | --- |
| AUC of $Lp$ | Correlation | 0.065 | -0.011 | 0.021 | -0.093 | -0.237 | -0.188 |
|  | P values | 0.824 | 0.969 | 0.943 | 0.752 | 0.414 | 0.503 |
| AUC of $Cp$ | Correlation | 0.281 | -0.077 | -0.267 | 0.227 | -0.234 | 0.054 |
|  | P values | 0.331 | 0.778 | 0.355 | 0.436 | 0.420 | 0.847 |
| AUC of $\gamma$ | Correlation | -0.215 | 0.232 | 0.256 | 0.081 | 0.145 | 0.328 |
|  | P values | 0.461 | 0.388 | 0.378 | 0.782 | 0.621 | 0.233 |
| AUC of $\lambda$ | Correlation | -0.106 | 0.084 | 0.094 | 0.063 | -0.348 | 0.055 |
|  | P values | 0.719 | 0.758 | 0.749 | 0.830 | 0.223 | 0.845 |
| AUC of $\sigma$ | Correlation | -0.168 | 0.200 | 0.23 | 0.054 | 0.219 | 0.292 |
|  | P values | 0.567 | 0.458 | 0.429 | 0.856 | 0.451 | 0.290 |
| AUC of $E_{glob}$ | Correlation | -0.104 | 0.042 | 0.044 | 0.104 | 0.236 | 0.221 |
|  | P values | 0.723 | 0.878 | 0.880 | 0.723 | 0.416 | 0.428 |
| AUC of $E_{loc}$ | Correlation | 0.261 | 0.008 | -0.231 | 0.424 | -0.175 | 0.318 |
|  | P values | 0.367 | 0.975 | 0.428 | 0.131 | 0.549 | 0.248 |

Note: * p<0.05 ** p<0.01.

**Table S5.** Correlation analysis between nodal properties of MBNs of NMOSD patients and clinical assessments (with age and gender as covariates).

|  | | DD | BPF | EDSS | PASAT | MFIS | MMSE |
| --- | --- | --- | --- | --- | --- | --- | --- |
| Relationship between nodal degree and clinical variable | | | | | | | |
| AAL19 | Correlation | 0.021 | -0.043 | 0.049 | -0.028 | 0.468 | 0.347 |
|  | P values | 0.944 | 0.875 | 0.868 | 0.924 | 0.091 | 0.204 |
| AAL24 | Correlation | 0.288 | -0.236 | -0.363 | 0.021 | -0.068 | -0.537 |
|  | P values | 0.318 | 0.378 | 0.202 | 0.943 | 0.817 | 0.039 |
| AAL26 | Correlation | 0.150 | -0.366 | -0.495 | 0.310 | -0.520 | -0.122 |
|  | P values | 0.609 | 0.163 | 0.072 | 0.281 | 0.057 | 0.666 |
| AAL60 | Correlation | -0.346 | -0.201 | 0.219 | -0.489 | 0.163 | 0.181 |
|  | P values | 0.226 | 0.456 | 0.452 | 0.076 | 0.578 | 0.519 |
| AAL69 | Correlation | -0.036 | -0.179 | 0.381 | -0.312 | 0.313 | -0.162 |
|  | P values | 0.902 | 0.508 | 0.179 | 0.277 | 0.276 | 0.565 |
| AAL36 | Correlation | -0.219 | 0.245 | 0.089 | 0.412 | 0.132 | 0.497 |
|  | P values | 0.452 | 0.361 | 0.762 | 0.144 | 0.654 | 0.059 |
| AAL56 | Correlation | -0.053 | 0.404 | -0.332 | 0.112 | -0.093 | 0.019 |
|  | P values | 0.858 | 0.121 | 0.247 | 0.704 | 0.752 | 0.946 |
| AAL66 | Correlation | -0.170 | 0.165 | -0.059 | 0.546 | -0.368 | 0.177 |
|  | P values | 0.560 | 0.541 | 0.840 | 0.043* | 0.196 | 0.528 |
| AAL76 | Correlation | -0.296 | 0.543 | 0.094 | 0.199 | 0.298 | 0.485 |
|  | P values | 0.305 | 0.030* | 0.750 | 0.495 | 0.300 | 0.067 |
| AAL89 | Correlation | -0.309 | 0.214 | -0.520 | 0.394 | -0.364 | 0.058 |
|  | P values | 0.282 | 0.426 | 0.057 | 0.163 | 0.201 | 0.839 |
| Relationship between nodal betweenness and clinical variable | | | | | | | |
| AAL10 | Correlation | 0.059 | -0.029 | 0.234 | -0.192 | 0.437 | 0.005 |
|  | P values | 0.841 | 0.916 | 0.421 | 0.510 | 0.119 | 0.986 |
| AAL23 | Correlation | -0.177 | 0.214 | -0.106 | -0.213 | 0.448 | 0.18 |
|  | P values | 0.545 | 0.426 | 0.717 | 0.465 | 0.108 | 0.521 |
| AAL27 | Correlation | 0.206 | 0.051 | 0.421 | -0.582 | 0.558 | -0.322 |
|  | P values | 0.480 | 0.852 | 0.133 | 0.029* | 0.038* | 0.243 |
| AAL38 | Correlation | -0.061 | 0.467 | 0.382 | -0.089 | 0.056 | -0.078 |
|  | P values | 0.836 | 0.068 | 0.178 | 0.762 | 0.849 | 0.781 |
| AAL41 | Correlation | -0.014 | 0.072 | -0.246 | 0.260 | -0.468 | -0.014 |
|  | P values | 0.961 | 0.790 | 0.396 | 0.370 | 0.092 | 0.961 |
| AAL52 | Correlation | 0.692 | -0.213 | -0.110 | -0.285 | 0.236 | -0.498 |
|  | P values | 0.006** | 0.428 | 0.708 | 0.324 | 0.416 | 0.059 |
| AAL81 | Correlation | -0.266 | -0.005 | -0.156 | -0.166 | 0.277 | -0.07 |
|  | P values | 0.357 | 0.985 | 0.595 | 0.571 | 0.337 | 0.805 |
| AAL21 | Correlation | 0.325 | 0.258 | 0.315 | -0.265 | 0.730 | 0.019 |
|  | P values | 0.257 | 0.335 | 0.272 | 0.360 | 0.003** | 0.945 |
| AAL44 | Correlation | 0.558 | -0.104 | -0.210 | 0.094 | -0.153 | -0.435 |
|  | P values | 0.038* | 0.701 | 0.470 | 0.749 | 0.602 | 0.105 |
| Relationship between nodal efficiency and clinical variable | | | | | | | |
| AAL10 | Correlation | -0.167 | -0.173 | -0.020 | 0.238 | -0.065 | 0.249 |
|  | P values | 0.567 | 0.521 | 0.946 | 0.412 | 0.826 | 0.371 |
| AAL19 | Correlation | 0.001 | 0.121 | -0.127 | -0.025 | 0.502 | 0.431 |
|  | P values | 0.998 | 0.654 | 0.665 | 0.933 | 0.067 | 0.108 |
| AAL24 | Correlation | 0.248 | -0.049 | -0.458 | 0.083 | -0.137 | -0.4 |
|  | P values | 0.392 | 0.858 | 0.100 | 0.778 | 0.640 | 0.14 |
| AAL26 | Correlation | 0.048 | -0.167 | -0.557 | 0.359 | -0.585 | -0.052 |
|  | P values | 0.871 | 0.536 | 0.038* | 0.208 | 0.028* | 0.853 |
| AAL52 | Correlation | 0.549 | 0.008 | 0.059 | -0.393 | 0.262 | -0.438 |
|  | P values | 0.042* | 0.977 | 0.841 | 0.165 | 0.365 | 0.102 |
| AAL60 | Correlation | -0.358 | -0.170 | 0.188 | -0.499 | 0.116 | 0.195 |
|  | P values | 0.208 | 0.528 | 0.520 | 0.069 | 0.692 | 0.486 |
| AAL69 | Correlation | -0.039 | -0.133 | 0.342 | -0.306 | 0.272 | -0.173 |
|  | P values | 0.895 | 0.624 | 0.231 | 0.287 | 0.346 | 0.538 |
| AAL56 | Correlation | -0.024 | 0.447 | -0.385 | 0.069 | -0.080 | -0.055 |
|  | P values | 0.935 | 0.082 | 0.174 | 0.814 | 0.787 | 0.845 |
| AAL66 | Correlation | -0.206 | 0.243 | -0.147 | 0.568 | -0.400 | 0.214 |
|  | P values | 0.480 | 0.364 | 0.616 | 0.034* | 0.156 | 0.445 |

Note: * p<0.05 ** p<0.01

**Table S6.** Correlation analysis between nodal properties of FBNs of NMOSD patients and clinical assessments (with age and gender as covariates).

|  | | DD | BPF | EDSS | PASAT | MFIS | MMSE |
| --- | --- | --- | --- | --- | --- | --- | --- |
| Nodal degree corrected with clinical variable | | | | | | | |
| AAL6 | Correlation | -0.133 | -0.133 | 0.092 | -0.271 | -0.227 | -0.226 |
|  | P values | 0.651 | 0.624 | 0.755 | 0.348 | 0.436 | 0.417 |
| AAL23 | Correlation | 0.331 | -0.102 | 0.426 | -0.418 | 0.109 | -0.454 |
|  | P values | 0.248 | 0.707 | 0.129 | 0.137 | 0.711 | 0.089 |
| AAL26 | Correlation | -0.557 | 0.448 | 0.115 | 0.120 | -0.345 | 0.432 |
|  | P values | 0.039* | 0.082 | 0.695 | 0.683 | 0.227 | 0.107 |
| AAL42 | Correlation | -0.165 | 0.104 | 0.196 | 0.082 | 0.079 | 0.583 |
|  | P values | 0.572 | 0.702 | 0.501 | 0.780 | 0.788 | 0.023* |
| AAL90 | Correlation | 0.059 | -0.155 | -0.462 | 0.233 | -0.385 | -0.022 |
|  | P values | 0.842 | 0.566 | 0.096 | 0.422 | 0.174 | 0.937 |
| AAL18 | Correlation | 0.267 | -0.129 | 0.043 | 0.363 | 0.084 | 0.199 |
|  | P values | 0.356 | 0.633 | 0.885 | 0.202 | 0.776 | 0.478 |
| AAL21 | Correlation | -0.458 | 0.342 | -0.200 | 0.372 | -0.254 | 0.358 |
|  | P values | 0.100 | 0.194 | 0.493 | 0.191 | 0.381 | 0.191 |
| AAL61 | Correlation | -0.244 | 0.205 | -0.387 | -0.114 | -0.342 | -0.109 |
|  | P values | 0.401 | 0.447 | 0.172 | 0.699 | 0.232 | 0.700 |
| AAL81 | Correlation | -0.103 | 0.122 | -0.058 | -0.165 | 0.089 | -0.353 |
|  | P values | 0.725 | 0.654 | 0.844 | 0.573 | 0.762 | 0.197 |
| Nodal betweenness corrected with clinical variable | | | | | | | |
| AAL69 | Correlation | -0.187 | -0.210 | 0.415 | -0.299 | 0.318 | 0.310 |
|  | P values | 0.522 | 0.435 | 0.140 | 0.298 | 0.268 | 0.260 |
| AAL75 | Correlation | 0.333 | -0.083 | 0.185 | -0.029 | -0.006 | -0.189 |
|  | P values | 0.245 | 0.761 | 0.526 | 0.922 | 0.983 | 0.500 |
| AAL90 | Correlation | -0.284 | -0.136 | -0.191 | 0.45 | -0.433 | 0.400 |
|  | P values | 0.326 | 0.615 | 0.513 | 0.107 | 0.122 | 0.139 |
| AAL3 | Correlation | -0.019 | 0.405 | -0.309 | 0.251 | -0.082 | -0.193 |
|  | P values | 0.948 | 0.120 | 0.282 | 0.387 | 0.78 | 0.491 |
| AAL21 | Correlation | -0.643 | 0.511 | -0.331 | 0.253 | -0.345 | 0.290 |
|  | P values | 0.013* | 0.043* | 0.248 | 0.384 | 0.228 | 0.295 |
| AAL29 | Correlation | -0.382 | 0.020 | 0.108 | 0.193 | -0.455 | 0.290 |
|  | P values | 0.178 | 0.942 | 0.714 | 0.508 | 0.102 | 0.295 |
| AAL73 | Correlation | -0.124 | -0.123 | 0.028 | 0.222 | 0.152 | 0.204 |
|  | P values | 0.673 | 0.649 | 0.924 | 0.446 | 0.604 | 0.465 |
| Nodal efficiency corrected with clinical variable | | | | | | | |
| AAL6 | Correlation | -0.124 | -0.125 | 0.057 | -0.25 | -0.209 | -0.243 |
|  | P values | 0.673 | 0.644 | 0.846 | 0.388 | 0.473 | 0.076 |
| AAL23 | Correlation | 0.349 | -0.117 | 0.491 | -0.425 | 0.174 | 0.475 |
|  | P values | 0.222 | 0.665 | 0.074 | 0.129 | 0.553 | 0.074 |
| AAL26 | Correlation | -0.598 | 0.466 | 0.097 | 0.132 | -0.283 | 0.475 |
|  | P values | 0.024* | 0.069 | 0.740 | 0.652 | 0.327 | 0.074 |
| AAL90 | Correlation | 0.06 | -0.150 | -0.504 | 0.275 | -0.348 | 0.070 |
|  | P values | 0.838 | 0.579 | 0.066 | 0.342 | 0.222 | 0.806 |
| AAL18 | Correlation | 0.221 | -0.093 | 0.073 | 0.317 | 0.186 | 0.276 |
|  | P values | 0.448 | 0.732 | 0.805 | 0.269 | 0.525 | 0.319 |
| AAL21 | Correlation | -0.412 | 0.290 | -0.05 | 0.456 | -0.169 | 0.474 |
|  | P values | 0.144 | 0.276 | 0.866 | 0.101 | 0.563 | 0.074 |
| AAL61 | Correlation | -0.411 | 0.264 | -0.356 | -0.063 | -0.318 | -0.007 |
|  | P values | 0.145 | 0.323 | 0.211 | 0.830 | 0.268 | 0.979 |
| AAL81 | Correlation | 0.521 | 0.083 | -0.06 | 0.124 | 0.384 | 0.213 |
|  | P values | 0.056 | 0.759 | 0.840 | 0.672 | 0.175 | 0.446 |

Note: * p<0.05 ** p<0.01.

**Table S7**. Relevant brain regions for the classification analysis.

| No. | Nodal Properties | AAL | Regions | Nodal Properties |  | Regions |
| --- | --- | --- | --- | --- | --- | --- |
| Altered nodal of FBN | | | | Altered nodal of MBN | | |
| 1 | NB | 21 | Olfactory_L | NB | 10 | Frontal_Mid_Orb_R |
| 2 | NB | 29 | Insula_L | NB | 21 | Olfactory_L |
| 3 | NB | 3 | Frontal_Sup_L | NB | 23 | Frontal_Sup_Medial_L |
| 4 | NB | 69 | Paracentral_Lobule_L | NB | 27 | Rectus_L |
| 5 | NB | 73 | Putamen_L | NB | 38 | Hippocampus_R |
| 6 | NB | 75 | Pallidum_L | NB | 41 | Amygdala_L |
| 7 | NB | 90 | Temporal_Inf_R | NB | 44 | Calcarine_R |
| 8 | ND | 18 | Rolandic_Oper_R | NB | 52 | Occipital_Mid_R |
| 9 | ND | 21 | Olfactory_L | NB | 81 | Temporal_Sup_L |
| 10 | ND | 23 | Frontal_Sup_Medial_L | ND | 19 | Supp_Motor_Area_L |
| 11 | ND | 26 | Frontal_Med_Orb_R | ND | 24 | Frontal_Sup_Medial_R |
| 12 | ND | 42 | Amygdala_R | ND | 26 | Frontal_Med_Orb_R |
| 13 | ND | 6 | Frontal_Sup_Orb_R | ND | 36 | Cingulum_Post_R |
| 14 | ND | 61 | Parietal_Inf_L | ND | 56 | Fusiform_R |
| 15 | ND | 68 | Precuneus_R | ND | 60 | Parietal_Sup_R |
| 16 | NE | 81 | Temporal_Sup_L | ND | 66 | Angular_R |
| 17 |  |  |  | ND | 69 | Paracentral_Lobule_L |
| 18 |  |  |  | ND | 76 | Pallidum_R |
| 19 |  |  |  | ND | 89 | Temporal_Inf_L |
| 20 |  |  |  | NE | 10 | Frontal_Mid_Orb_R |
| 21 |  |  |  | NE | 52 | Occipital_Mid_R |

Abbreviations: AAL, automated anatomical atlas; AUC, area under the curve; FBN, functional brain network; MBN, morphological brain network; ROC, receiver operating characteristic curves; Nb, nodal betweenness; Nd, nodal degree; Ne, nodal efficiency.
